# Enantioselective HPLC Analysis to Assist the Chemical Exploration of Chiral Imidazolines

**DOI:** 10.3390/molecules25030640

**Published:** 2020-02-02

**Authors:** Bruno Cerra, Antonio Macchiarulo, Andrea Carotti, Emidio Camaioni, Ina Varfaj, Roccaldo Sardella, Antimo Gioiello

**Affiliations:** Department of Pharmaceutical Sciences, University of Perugia, Via Fabretti 48, 06123 Perugia, Italy; bruno.cerra@chimfarm.unipg.it (B.C.); antonio.macchiarulo@unipg.it (A.M.); andrea.carotti@unipg.it (A.C.); emidio.camaioni@unipg.it (E.C.); ina.varfaj@outlook.com (I.V.); antimo.gioiello@unipg.it (A.G.)

**Keywords:** cellulose-based chiral stationary phase, chiral imidazolines, enantiorecognition mechanism, hit-to-lead chemical exploration, reversed-phase conditions

## Abstract

In the present work, we illustrate the ability of high-performance liquid chromatography (HPLC) analysis to assist the synthesis of chiral imidazolines within our medicinal chemistry programs. In particular, a Chiralpak^®^ IB^®^ column containing cellulose tris(3,5-dimethylphenylcarbamate) immobilized onto a 5 μm silica gel was used for the enantioselective HPLC analysis of chiral imidazolines synthesized in the frame of hit-to-lead explorations and designed for exploring the effect of diverse amide substitutions. Very profitably, reversed-phase (RP) conditions succeeded in resolving the enantiomers in nine out of the 10 investigated enantiomeric pairs, with α values always higher than 1.10 and R_S_ values up to 2.31. All compounds were analysed with 50% (v) water while varying the content of the two organic modifiers acetonitrile and methanol. All the employed eluent systems were buffered with 40 mM ammonium acetate while the apparent pH was fixed at 7.5. Based on the experimental results, the prominent role of π-π stacking interactions between the substituted electron-rich phenyl groups outside of the polymeric selector and the complementary aromatic region in defining analyte retention and stereodiscrimination was identified. The importance of compound polarity in explaining the retention behaviour with the employed RP system was readily evident when a quantitative structure-property relationship study was performed on the retention factor values (k) of the 10 compounds, as computed with a 30% (v) methanol containing mobile phase. Indeed, good Pearson correlation coefficients of retention factors (r - log k_1st_ = −0.93; r - log k_2nd_ = −0.94) were obtained with a water solubility descriptor (Ali-logS). Interestingly, a *n*-hexane/chloroform/ethanol (88:10:2, *v*/*v*/*v*)-based non-standard mobile phase allowed the almost base-line enantioseparation (α = 1.06; R_S_ = 1.26) of the unique compound undiscriminated under RP conditions.

## 1. Introduction

Among the numerous chromatographic techniques available today for chiral analysis, high-performance liquid chromatography (HPLC) excels at this task due to its simplicity and effectiveness. Moreover, HPLC is an easy-to-use, versatile technique to assist synthesis in view of the wide array of chemicals detection and low implementation time, and also when integrated with flow synthesizers and high-throughput experimentation (HTE) systems. Thanks to the continuous technological advancements in the production of Chiral Stationary Phases (CSPs), HPLC-based methods have gotten faster and more effective for the enantiopurity assessment of chiral drugs [1,2,3,4,5]. To date, several hundred CSPs for HPLC applications are commercially available. However, it needs to be clearly stated that a universal CSP enabling the enantioseparation of all classes of racemic compounds is still missing. In some instances, selecting the proper CSP for the enantioseparation of a chiral compound can be rather difficult.

Cellulose and amylose tris(phenylcarbamates) and tris(benzoates) are actually the most versatile CSPs currently available [1,3,6]. Indeed, this family of CSPs has demonstrated to produce successful enantioseparations for most of the reported chiral chromatography applications [1,3,7,8,9]. Although the chiral recognition mechanism with these cellulose- and amylose-based chiral selectors are still a matter of profound debate, it is widely accepted that a sequence of “chiral grooves” hosting the polar carbamate or ester residues exists along the main chain of the helix, with the phenyl rings being projected outwards the polymer chain [1,3,6,10,11,12]. When mobile phases typical of the normal-phase (NP) elution mode are used, the analyte enantiomers can activate stereoselective interactions with the polar carbamate or ester groups via hydrogen bonding with the O atom and/or the NH and C=O groups, as well as dipole-dipole interaction with the C=O residue located within the above cavities [1,3,6,10,11,12]. Besides these polar contacts, π-π stacking interactions between the phenyl groups on the CSP and an aromatic region or an unsaturated portion of the solute may play some role in chiral recognition, especially when analyses are run in the reversed-phase (RP) mode [13,14,15,16,17]. In RP applications, the effect of other types of hydrophobic interactions, such as those involving the carbon backbone of the sugar moieties, can have a role as well.

One of the main advantages of polysaccharide-type CSPs is the “multimodal” nature of their chiral recognition capacity, which makes them compatible with all of the most relevant chromatographic mobile phase regimes. Even though polysaccharide-type CSPs are most commonly operated under NP conditions [1,3,6,10,11,12], employing mixtures of *n*-hexane with polar organic alcohol modifiers, these phases have also been shown to produce excellent enantioselective levels both with RP and polar organic (PO) or polar ionic (PI) eluent systems [13,14,15,16,17]. Moreover, with the new generation of “immobilized CSPs” where the chiral selector is covalently grafted onto a pre-functionalised solid support, eluent components previously prohibited with the first generation of polymeric phases can be also used, thus allowing the possibility to explore additional stereoselectivity profiles [11,18]. Very importantly, the lack of any restriction in terms of mobile phase composition and solubilization solvents strongly facilitates the direct injection of samples from synthesis media onto the columns by automated sampling systems.

**Figure 1:**
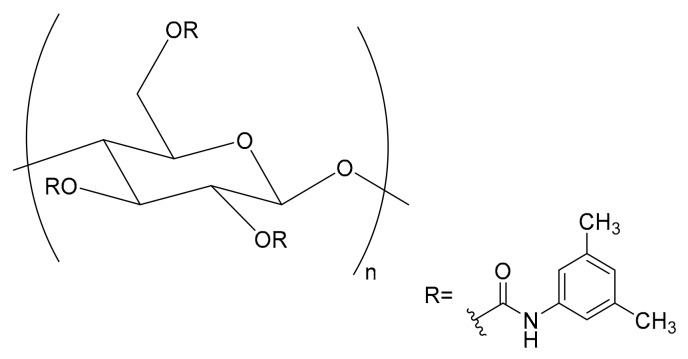
Chiral selector characterizing the Chiralpak^®^ IB^®^ stationary phase. The cellulose-based polymer is covalently immobilized to a pre-functionalized solid support.

**Figure 2:**
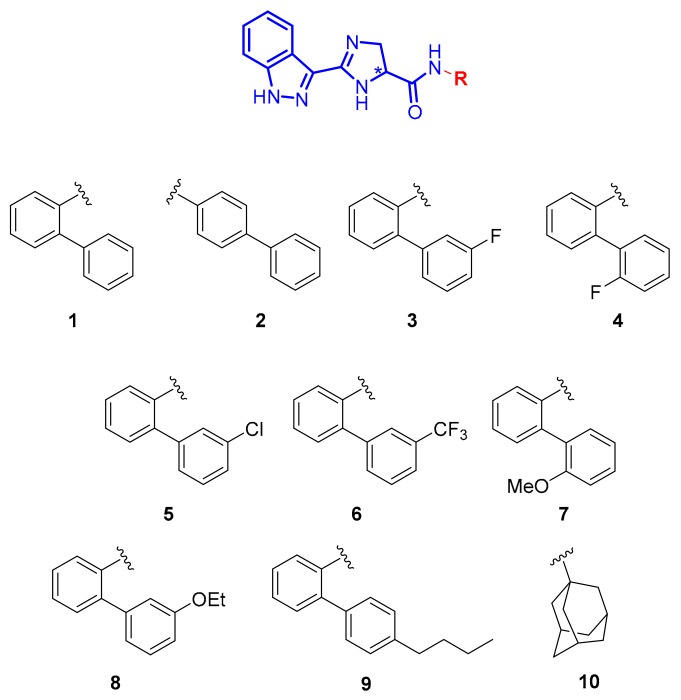
Compounds investigated in this study.

With the present work, we intend to demonstrate that the Chiralpak^®^ IB^®^ column (Figure 1) containing cellulose tris(3,5-dimethylphenylcarbamate) immobilized onto a 5 μm silica gel can be successfully used under RP conditions for the enantioselective HPLC analysis of chiral imidazolines. The compounds herein analysed were synthesized in the frame of a hit-to-lead exploration program aimed at evaluating the effect of diverse amide substitutions (Figure 2) [19] towards a metabolic nuclear receptor.

## 2. Results and Discussion

The imidazoline core was selected as a promising scaffold on the base of a screening campaign designed for the identification of a pool of commercially available compounds to be submitted for biological testing against a target of our interest. At the end of this process, a structure-based drug design approach was used to drive the synthesis of a small library of chiral imidazolines, including compounds 1–10. Once the compounds are assayed as racemates, the most active analogues would require a deeper investigation to assess the chiral contribution to the biological activity. As a consequence, to avail of enantioselective chromatography methods allowing to assess the enantiopurity of the compounds from enantioselective synthesis protocols is urgently needed.

### 2.1. Optimization of the Enantioselective HPLC Analysis

With the aim to obtain the enantioseparation of the investigated compounds (Figure 2) with the cellulose-based Chiralpak^®^ IB^®^ (Figure 1), chiral stationary phase (CSP) and eluent systems coherent with the most commonly applied normal phase (NP) conditions were initially used.

None of the tested *n*-hexane/alcohol combinations produced base-line separation of the investigated compounds. The alternative and less exploited reversed-phase (RP) mode of elution was thus explored. Running the analyses without any restriction in terms of mobile phase composition was made possible by the immobilized nature of the selected cellulose-based 3,5-dimethylphenyl carbamate-based material [11,13,18]. Although the immobilized polysaccharide-based CSPs can be conveniently employed under RP conditions, only a few application reports can be found in the literature [13,14,15,16,17]. Mostly during the last decades, several authors demonstrated that with this class of enantiodiscriminating material, chiral recognition is still possible even when eluent systems disfavouring H-bonding between selector and selectand units are used [13,14,15,16,17]. Under RP conditions, compatibility with mass spectrometer (MS) detection systems is favoured, while pump cavitation often caused by low-boiling points NP solvents is sensitively reduced [14]. Not less important, the use of aqueous mobile phases for RP applications is increasingly solicited based on their economic and environmental benefits, as well as to cope with solubility limitations often found in NP experiments.

In accordance to the proposed screening strategies for RP analysis with such type of CSPs [20], a mobile phase as similar as possible to that succeeding the achiral analysis on the same pool of compounds was initially assayed. However, the eluent composed of 40 mM NH_4_OAc/ACN (50:50, *v*/*v*; pwsH 7.5), did not favour the optimal performance by the polymer. Indeed, nine compounds (**1**–**9**) out of 10 experienced enantioseparation without getting resolution, while the enantiomers of 10 did co-elute at all.

With the objective of further improving the chromatographic performance in the RP mode of elution, focused modifications of the eluent composition were successively screened by keeping unaltered the buffer type and concentration as well as the eluent pwsH. To run the analyses at a pwsH around neutrality, it was necessary to preserve the chemical integrity of imidazoline heterocycles.

For all compounds, the peak resolution still missed when other binary water/ACN-based eluents were scrutinized. Interestingly, the progressive replacement of ACN with methanol (MeOH), while keeping constant the water content, sensitively improved both the thermodynamic and mostly the kinetic properties of the enantiorecognition system. Compound **10** did not experience enantioseparation in all the tested RP ternary mixtures. Not all samples were analysed with the same eluents, but for each, the investigation was stopped once obtained the desired result (namely the base-line resolution of the enantiomeric peaks). In Table 1, the chromatographic parameters (retention factors of the first and second eluted enantiomer k_1_ and k_2_, resolution R_S_ and enantioselectivity α) under optimal experimental conditions are reported, whilst the data achieved with all the other screened experimental settings are shown in Table S1 (Supplementary Materials).

The presence of certain amount of ACN was important due to the number of distinctive advantages of this organic modifier: low viscosity, low cut-off, and good eluotropic power for polysaccharide-based CSPs, among the others [20].

A MeOH content lower than 30% in volume was always maintained in order to avoid too high back-pressures and unpractical analysis times. Indeed, similarly to achiral RP analyses, rising the amount of MeOH produced for all compounds an increase in retention along with an improvement of both separation and resolution factors. This latter phenomenon, that is, improving chiral resolution with a longer permanence of the analytes in the column, is not so common in chiral chromatography: in many instances, retention times that are too long can cause an excessive increase in peak widths only partially counterbalanced by any benefits in terms of selectivity and efficiency [17].

**Figure 3:**
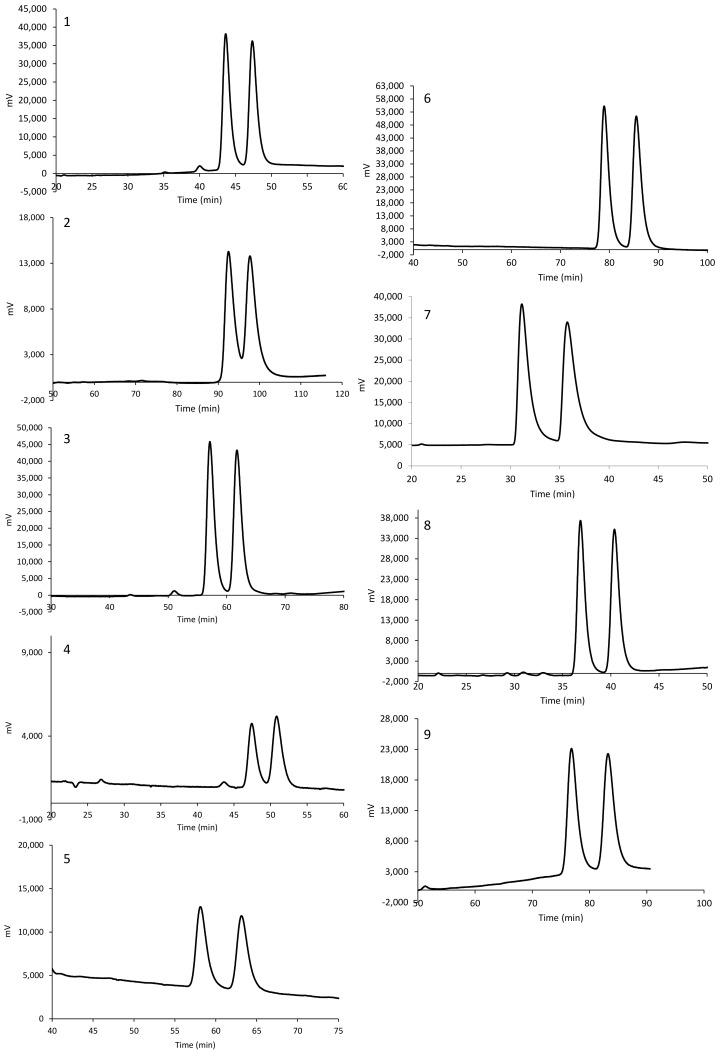
Chromatograms obtained for compounds **1**–**9** on Chiralpak IB under optimized RP conditions.

The chromatograms with the optimized RP conditions are shown in Figure 3 for compounds **1**–**9**. As evident from Figure 3 and the results listed in Table 1, only racemate 2 was not resolved under the tested RP conditions. The peculiar behaviour of this compound, with respect to its regioisomer 1, can be plausibly ascribed to a different selector-selectand association fit.

With the exception of compound **10**, all the other analytes were successfully enantiodiscriminated into a RP setting, thus indicating a key part of the biphenylic portion in the RP recognition event. Based on the predicted logP and logS values (with S standing for water solubility) listed in Table S2, compound **10** results in the most hydrophilic within the investigated analytes, irrespective of the in silico method applied for the estimation. This evidence readily suggests that a certain hydrophobicity is needed to obtain RP enantioselectivity of the investigated chiral imidazolines with the selected CSP.

**Figure 4:**
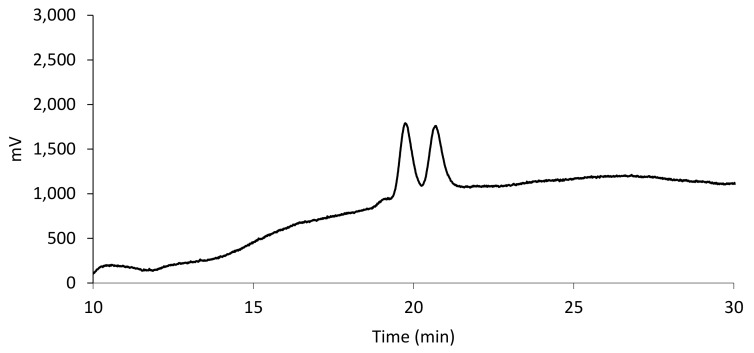
Chromatogram of compound **10**. Eluent: *n*-hexane/chloroform/EtOH (88:10:2, *v*/*v*/*v*), flow rate: 1.0 mL min^−1^, column temperature: 20 °C, wavelength of detection: 254 nm.

The difficulty to identify suitable RP conditions for compound **10** stimulated us to attempt its enantioresolution with the incorporation of “non-standard” solvents into NP eluents. Indeed, the immobilized nature of the selected CSP expands the solvent compatibility of the enantiodiscriminating material and, in turn, opens the way to previously unexplored selectivity profiles. Interestingly, a *n*-hexane/chloroform/EtOH (88:10:2, *v*/*v*/*v*) based mobile phase allowed the almost base-line separation (α = 1.06; R_S_ = 1.26) of enantiomers of racemate 10 within usable analysis time (Figure 4).

Evidently, the conformational alteration caused by the polymer immobilization that often leads to a reduced enantioseparation ability in NP environments [21,22] can be profitably modulated with a careful selection of the mobile phase composition. Accordingly, the use of chloroform induced peculiar conformational arrangements in both selector and analyte structure, and hence contributed to profitably rule the extent of complementarities of the intermolecular interactions between them.

### 2.2. Mechanism of Retention and Enantiorecognition Under RP Conditions

Plotting the retention factor and separation factor values against the MeOH content in the eluent revealed that the mechanisms of retention and enantiorecognition was not altered by the eluent composition. Indeed, the thermodynamic features of the selector-selectand association mechanism did not change with all the screened ternary eluent systems. In Figure S1 (Supplementary Materials), the chromatographic behaviour for compound **1** is exemplarily shown.

As far as the sample retention is concerned, a series of hypotheses can be formulated to account for the retention behaviour recorded with the screened mobile phases. First, in contrast to ACN, the absence of π−electrons in MeOH significantly reduces the ability by the eluent to realize dispersive interactions with the analyte enantiomers. Nonetheless, MeOH is recognized to form a strong associate with water which reduces its eluotropic power into a RP setting [23]. Further hypotheses can be argued if the issue is approached from the standpoint of the CSP. The H-bond and dipole-dipole interaction sites, primarily expressed into a “classical” NP environment by the carbamate N-H and C=O groups inside the helical grooves, are strongly solvated when RP elution conditions are used [20,24]. The chance of saturation of the above sites by the eluent molecules significantly increases as the MeOH content in the eluent is made to increase. Therefore, as a matter of fact, the strong solvation of the inner ravines of the cellulose-based polymer significantly modifies the selector-selectand interaction mechanism [20]. In this framework, the rather irrelevant retention power by H-bond and dipole-dipole contacts was highlighted with a further increase of the water content in the eluent. Exemplary is the case of compound **3** for which a 5% water volume increment at expense of ACN (40 mM NH_4_OAc/ACN/MeOH 55:15:30, *v*/*v*/*v*) elongated the analyte retention (Figure S2, Supplementary Materials), while contemporarily producing a slight improvement of R_S_ (equal to 1.98). Interestingly, a negligible variation was still observed in terms of α, meaning that stereoselective contacts are not altered by such an eluent modification.

On the basis of all the above considerations, the π−π interactions between the substituted electron-rich phenyl groups outside of the polymeric selector and the complementary aromatic region of solute should play a prominent role in the frame of the enantiorecognition process [24,25,26,27,28,29,30,31]. Moreover, the effect of other types of hydrophobic interactions, viz those with the carbon backbone of the sugar moieties, can have a role as well in tuning the analyte retention.

The improvement of the chromatographic performance with an increase of MeOH content clearly supports the above assumption coping with a focal part of hydrophobic interactions during the selector-selectand association. Indeed, ACN generally produces a stronger eluotropic effect than MeOH thanks to the higher ability by the former to enter dispersive interactions [23]. Moreover, ACN is also recognized to be a stronger competitor towards π−π interactions through its unsaturated triple bond [32], capable to reduce the chance (and strength) of the selector-selectand π−π stacking-based associations.

The different chromatographic behaviour displayed by the investigated compounds can be plausibly ascribed to differences in their electronic characteristics. Indeed, different substituents producing specific electron-donating or -withdrawing effects are present on the diphenyl moiety of compounds **1**–**9**, thus promoting peculiar enantioselective selector-analyte associations involving their aromatic regions. Data reported in Table 1 seems to suggest that compounds **7**–**9**, carrying an electron-donating moiety, can be profitably enantioresolved with a relatively lower MeOH content than compounds **3**–**6** having an electron-withdrawing substituent on the R group.

On the basis of the different chromatographic behaviour of compounds **1** and **2**, and in analogy to elutions under NP conditions, a conformationally driven enantiorecognition mechanism [33] can be conjectured also in the case of the described RP analyses. In this frame, hydrophobic contacts can be presumed as fundamental for the initial solute-CSP interaction (“leading-dominating interactions” [24]), followed by conformational adjustments and differential inclusion phenomena of other regions of the analyte inside the “calyxes” along the polysaccharide chain (“secondary-supporting” interactions [24]). Further, the interaction between the sample enantiomer and adjacent 3,5-dimethylphenyl units cannot be ruled out.

The key role of compound polarity in defining the retention time with the employed RP system was readily evident when a quantitative structure-property relationship (QSPR) study was performed on the retention factor values (k) of the 10 compounds as computed with a 30% (v) MeOH mobile phase.

**Figure 5:**
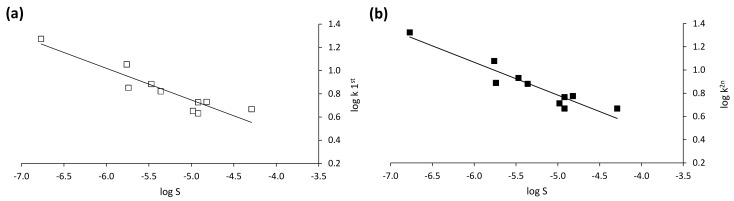
Relationships between the logarithm of aqueous solubility (log S) and the logarithm of analyte retention factor for (**a**) the first eluted peak (log k_1st_) and (**b**) the second eluted peak (log k_2nd_) of each enantiomer pair. The rightmost point in the two plots refers to compound **10**. Experimental conditions: column, Chiralpak^®^ IB^®^; mobile phase, water/MeOH/ACN (50:30:20, *v*/*v*/*v*) (with 40 mM NH_4_OAc, pwsH 7.5); flow rate, 0.4 mL min^−1^; column temperature, 35 °C; wavelength of detection: 254 nm.

As shown in Table 2, good Pearson correlation coefficients (r) of retention factors (r - logk_1st_ = −0.93; r - log k_2nd_ = −0.94) with *p* < 0.001 were obtained with a water solubility descriptor (Ali-logS) [34], meaning the higher the compound aqueous solubility, the lower its residence time in the column (Figure 5). This result is consistent with a typical RP mechanism in which compounds are eluted according to their polarity extent. For compound **10**, log k value is the same for both enantiomers. However, this point was also added in the QSPR study, since retention is often mostly influenced by non-enantioselective selector-selectand interactions.

## 3. Material and Methods

### 3.1. Chemicals

With the exception of analytes, all chemicals were of analytical reagent grade and used as received. Ethanol (EtOH), methanol (MeOH), acetonitrile (ACN), *n*-hexane, and chloroform were purchased from Sigma–Aldrich (Milan, Italy). Ammonium acetate, acetic acid, and ammonia solution were purchased from Carlo Erba (Milan, Italy). HPLC grade water was obtained from a tandem Milli-Ro/Milli-Q apparatus (Millipore, Bedford, MA, USA). All the employed RP mobile phases were filtered through a 0.22 μm Millipore filter (Bedford, MA, USA). Both RP and NP eluents were degassed with 20 min sonication before use. In all cases, analytes to be injected were solubilized in the selected mobile phase. All investigated compounds (**1**–**10**) were synthesized in our laboratory [19].

### 3.2. Instrumentations

All the HPLC experiments were carried out on a Shimadzu (Kyoto, Japan) Class-VP equipped with a EZ Start chromatography data software, a LC-10 ATVP pump, a SCL-10AVP system controller, a FCV-10ALVP low pressure gradient formation unit, a DGU-14A online degasser, and a Rheodyne 7725i injector (Rheodyne, Cotati, CA, USA) with a 20 mL stainless steel loop. The Chiralpak^®^ IB^®^ [250 mm x 4.6 mm I.D. containing cellulose tris(3,5-dimethylphenylcarbamate) immobilized onto a 5 μm silica gel] was purchased from Chiral Technologies (West Chester, PA, USA) and used as the chiral column for all the analyses. The column was used only after previous conditioning with the selected mobile phase with at least 20 void volumes. While RP analyses were carried always out at a 0.4 mL min^−1^, those in the NP mode were run at a 1.0 mL min^−1^ unless otherwise stated. The eluents for RP analyses were prepared by dissolving 20 mM NH_4_OAc in the selected organic modifier/HPLC grade water mobile phase. Then, the apparent pH [pwsH, which is the one measured in the employed hydro-organic mobile phase(s) while the calibration of the pH system was done in water (w)] was adjusted to 7.5 with NH_4_OH or AcOH. Column temperature was controlled through a Grace (Sedriano, Italy) heater/chiller (Model 7956R) thermostat. Sodium nitrite was used as the unretained marker for dead times determinations. A conventional spectrophotometric detector was used, and the UV detection wavelength was set at 254 nm for all the analyses.

### 3.3. Molecular Modelling and Statistical Analysis.

Compounds **1**–**10** were drawn using Maestro software package (Schrödinger Release 2019-1: Maestro, Schrödinger, LLC, New York, NY, USA, 2019) and prepared using the Ligprep utility (Schrödinger Release 2019-1: LigPrep, Schrödinger, LLC, New York, NY, USA, 2019). The resulting structures were then imported into Canvas (Schrödinger Release 2019-1: Canvas, Schrödinger, LLC, New York, NY, 2019) for calculation of *n*-octanol/water partition coefficient descriptors including AlogP and QPlogPo/w. Additional *n*-octanol/water partition coefficient descriptors (ilogP, XlogP3, WlogP, MlogP, SilicosIT-logP) and water solubility descriptors (ESOL-logS, Ali-logS, SilicosIT-logSw) were calculated using Swiss-ADME tool [34]. Statistical analyses were performed with the aid of the open source software CRAN-R, version 3.4.0 [35].

## 4. Conclusions

Shortening timelines for chiral drug discovery and development requires, inter alia, efficient chromatographic enantioseparation methods. Indeed, as chiral chemical probes and drug candidates are emerging from laboratories at an extraordinary rate, the necessity of suitable analytical techniques and methods to quickly faced with the massive production of chiral therapeutics is mandatory. In the present work, a Chiralpak^®^ IB^®^ column containing cellulose tris(3,5-dimethylphenylcarbamate) immobilized onto a 5 μm silica gel was used under RP conditions for the enantioselective HPLC analysis of chiral imidazolines synthesized in the frame of hit-to-lead explorations and designed for exploring the effect of diverse amide substitutions. Very profitably, the scarcely applied RP conditions with 40 mM NH_4_OAc/ACN/MeOH-based eluent systems succeeded in resolving the enantiomers of nine out of the 10 investigated pairs. In spite of the long runtime required to get the enantioseparation of some of the investigated compounds (especially **2**, **6**, and **9**), the proposed RP elution methods hold several advantages, including the high compatibility with mass spectrometry detectors, which render the methods also suitable for bioanalytical assays. The use of aqueous mobile phases fully complies with the paradigms of green chemistry. The use of a column with smaller dimensions (in terms of length and particle size) will be evaluated to reduce the runtime of the most identified bioactive species.

In order to gain a deeper insight into the main force(s) ruling the retention mechanism of the investigated chiral imidazolines in the employed chromatographic setting, a quantitative structure-property relationship study was performed. Consistent with a typical RP mechanism, a trend of elution in line with the analyte polarity was highlighted.

In conclusion, with the present contribution, we have demonstrated that polysaccharide-based CSPs in combination with RP mobile phases can be successfully used to assist enantioselective synthesis protocols, thereby opening the way to future integration with flow synthesizers and to the set-up of automated systems for enantioselective synthesis and medicinal chemistry explorations. As previously stated, the biological assays which are intended to be preliminarily performed with the racemates of compounds **1**–**10**, should reveal the most interesting ones to be further investigated in terms of the effect of stereochemistry on biological action. Accordingly, dedicated asymmetric synthesis protocols will be developed and results will be useful to establish the enantiomeric elution order (EEO) with the identified best chromatographic conditions. Along this line, for this (or these) compound(s), the enantioselective chromatography methods will be validated according to ICH guidelines.

## Figures and Tables

**Table 1:** Chromatographic data obtained for compounds **1**–**10** on Chiralpak IB under optimized RP conditions. Mobile phase: ACN/MeOH/40 mM NH_4_OAc (pwsH 7.5); flow rate: 0.4 mL min^−1^; column temperature: 35 °C; wavelength of detection: 254 nm.

Compound	% MeOH (v)	Selected Chromatographic Parameters			
		k_1_	k_2_	R_S_	α
**1**	25	4.99	5.50	1.94	1.10
**2**	25	11.28	11.97	1.21	1.06
**3**	30	6.85	7.48	1.90	1.09
**4**	30	5.51	5.97	1.48	1.08
**5**	25	6.98	7.67	2.06	1.10
**6**	30	9.55	10.43	1.96	1.09
**7**	20	3.49	3.94	2.28	1.13
**8**	15	4.06	4.54	2.31	1.12
**9**	15	9.84	10.74	2.16	1.09
**10**	Co-elution with all the tested conditions				

**Table 2:** Pearson correlation coefficients (*r*) of retention factors (logk_1st_, logk_2nd_), *n*-octanol/water partition coefficient (logP) and aqueous solubility (S) descriptors. Statistical significance is reported within parentheses.

Descriptor	*r* - logk_1st_	*r* - logk_2nd_
AlogP	0.45 (*p* = 0.20)	0.50 (*p* = 0.14)
QlogPo/w	0.41 (*p* = 0.23)	0.48 (*p* = 0.16)
ilogP	0.23 (*p* = 0.53)	0.25 (*p* = 0.49)
XlogP3	0.46 (*p* = 0.18)	0.50 (*p* = 0.14)
WlogP	0.70 (*p* = 0.03)	0.75 (*p* = 0.01)
MlogP	0.53 (*p* = 0.11)	0.58 (*p* = 0.08)
SilicosIT-logP	0.39 (*p* = 0.26)	0.46 (*p* = 0.18)
ESOL-logS	−0.80 (*p* = 0.005)	−0.83 (*p* = 0.003)
Ali-logS	−0.93 (*p* < 0.001)	−0.94 (*p* < 0.001)
SilicosIT-logSw	−0.39 (*p* = 0.27)	−0.45 (*p* = 0.19)

## Supplementary Materials

The following are available online, Table S1: chromatographic performances achieved with all the screened experimental setting for compounds **1**–**10** in the RP-mode of elution; Table S2: Values of the computational descriptors calculated in the study; Figure S1: variation of retention and separation factor values with the amount of MeOH for compound **1**; Figure S2: chromatographic trace of compound **3**.
