# Supplementary material for: Enantioselective HPLC Analysis to Assist the Chemical Exploration of Chiral Imidazolines
Source: Molecules. 2020 Feb 2;25(3):640. doi: 10.3390/molecules25030640 (PMC7036806; doi:10.3390/molecules25030640)

SUPPORTING INFORMATION

Enantioselective HPLC Analysis to Assist the Chemical Exploration of Chiral Imidazolines

Bruno Cerra, Antonio Macchiarulo, Andrea Carotti, Emidio Camaioni, Ina Varfaj, Roccaldo Sardella*, Antimo Gioiello

Department of Pharmaceutical Sciences, University of Perugia, Via Fabretti 48, 06123, Perugia, Italy; bruno.cerra@chimfarm.unipg.it (B.C.); antonio.macchiarulo@unipg.it (A.M.); andrea.carotti@unipg.it (A.C.); emidio.camaioni@unipg.it (E.C.); ina.varfaj@outlook.com (I.V.); antimo.gioiello@unipg.it (A.G.).

*Correspondence: roccaldo.sardella@unipg.it (R.S.)

Table S1. Chromatographic data achieved with all the screened experimental setting for compounds 1–10 in the RP-mode of elution. Column: Chiralpak IB; mobile phase: ACN/MeOH/40 mM NH_4OAc (40/10/50-20/30/50, v/v/v), $^{s}_{w}pH$ 7.5; flow rate: 0.4 mL min^{-1}; column temperature: 35 ℃; wavelength of detection: 254 nm.

Compound	% MeOH (v)	Selected chromathographic parameters			
		k_1	k_2	R_S	α
**1**	10	2.84	3.06	1.34	1.08
	15	3.32	3.61	1.55	1.09
	20	4.15	4.54	1.78	1.10
	**25**	**4.99**	**5.50**	**1.94**	**1.10**
	30	5.36	5.97	n.c.[a]	1.11
**2**	10	5.18	5.18	n.c.[a]	1.00
	15	6.43	6.43	n.c.[a]	1.00
	20	8.79	8.95	n.c.[a]	1.02
	**25**	**11.28**	**11.97**	**1.21**	**1.06**
	30	17.45	18.10	0.90	1.04
**3**	10	2.80	2.98	1.03	1.06
	15	3.34	3.57	1.29	1.07
	20	4.18	4.50	1.45	1.08
	25	5.03	5.45	1.65	1.08
	**30**	**6.85**	**7.48**	**1.90**	**1.09**
**4**	10	2.44	2.59	0.97	1.06
	15	2.79	2.97	1.11	1.07
	20	3.45	3.69	1.20	1.07
	25	4.10	4.42	1.30	1.08
	**30**	**5.51**	**5.97**	**1.48**	**1.08**
**5**	10	3.75	4.03	1.11	1.07
	15	4.66	5.05	1.59	1.09
	20	5.82	6.35	1.83	1.09
	**25**	**6.98**	**7.67**	**2.06**	**1.10**
	30	7.66	8.53	n.c.[a]	1.11
**6**	10	3.72	3.95	1.11	1.06
	15	4.32	4.61	1.29	1.07
	20	5.53	5.94	1.51	1.07
	25	6.74	7.29	1.70	1.08
	**30**	**9.55**	**10.43**	**1.96**	**1.09**
**7**	10	2.39	2.65	1.78	1.11
	15	2.83	3.16	2.02	1.12
	**20**	**3.49**	**3.94**	**2.28**	**1.13**
	30	4.48	5.17	n.c.[a]	1.15
**8**	10	3.48	3.86	2.02	1.11
	**15**	**4.06**	**4.54**	**2.31**	**1.12**
	30	6.63	7.60	n.c.[a]	1.15
**9**	10	7.81	8.45	1.83	1.08
	**15**	**9.84**	**10.74**	**2.16**	**1.09**
	30	18.74	21.10	n.c.[a]	1.13
**10**	Co-elution with all the tested conditions				

[a]Not calculated by the software.

Table S2. Values of the computational descriptors calculated in the study.

Compound	AlogP	QPlogPo/w	ilogP	XlogP3	WlogP	MlogP	SilicosIT-log P	ESOL-log S	Ali-logS	SilicosIT-logSw
**1**	3.34	4.40	2.45	3.41	2.63	3.06	4.31	-4.56	-4.82	-8.57
**2**	4.01	4.48	2.78	4.32	3.02	3.28	4.41	-5.02	-5.76	-7.48
**3**	3.55	4.68	2.64	3.51	3.19	3.44	4.72	-4.72	-4.92	-8.83
**4**	3.32	4.54	2.52	3.38	2.64	2.74	4.36	-4.72	-4.92	-8.83
**5**	3.55	4.64	2.28	3.51	3.19	3.44	4.72	-5.15	-5.47	-9.15
**6**	3.67	4.95	2.68	3.75	3.03	2.95	4.75	-5.41	-5.74	-9.39
**7**	4.01	4.89	2.35	4.04	3.29	3.54	4.94	-4.63	-4.98	-8.67
**8**	5.20	5.81	3.11	5.29	3.98	3.89	6.01	-4.86	-5.36	-9.06
**9**	4.28	5.38	2.51	4.3	4.81	3.85	5.36	-5.83	-6.77	-10.12
**10**	2.39	3.14	2.25	2.9	1.6	2.8	3.38	-3.9	-4.29	-5.61

Figure S1. **Variation of (a) retention and (b) separation factor values with the amount of MeOH in the ternary RP eluent, for compound 1. Column: Chiralpak IB; mobile phase: ACN/MeOH/40 mM NH_4OAc (40/10/50-20/30/50, v/v/v; $^{s}_{w}pH$ 7.5); flow rate: 0.4 mL min^{-1}; column temperature: 35 ℃; wavelength of detection: 254 nm.**

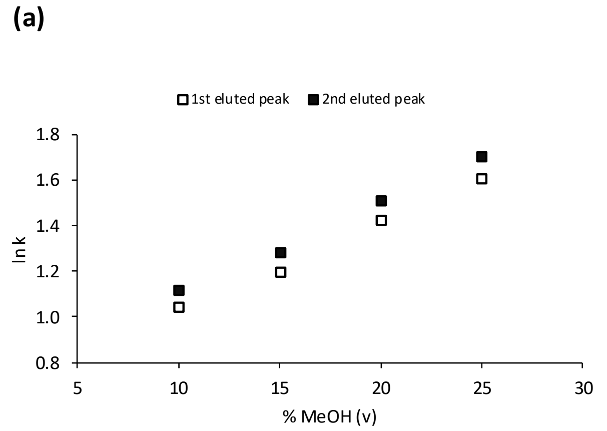

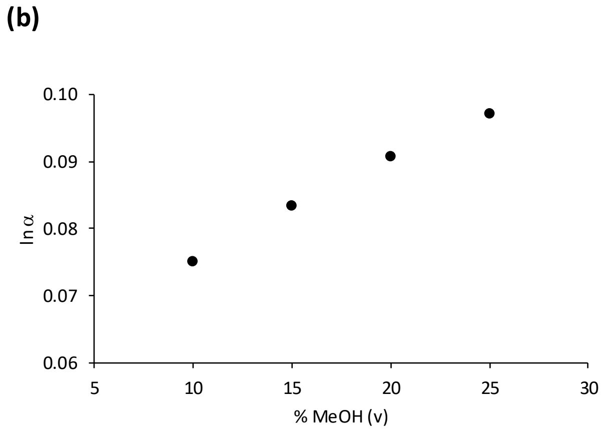

Figure S2. Chromatogram of compound **3**. Eluent: **40 mM NH_4OAc/ACN/MeOH** (55:15:30, v/v/v; ${}_{w}^{s}pH$ 7.5), flow rate: 0.4 mL min^{-1}, column temperature: 35 ℃, wavelength of detection: 254 nm.

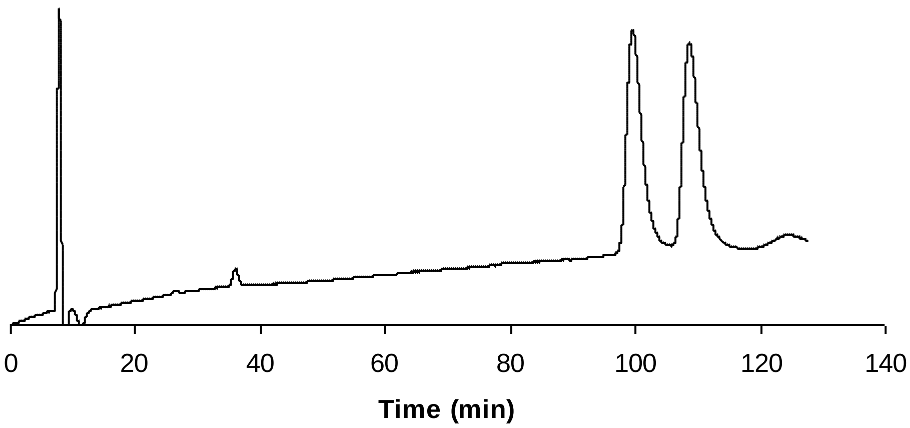

Supplement: Supplementary file 1 [file molecules-25-00640-s001.pdf]
